# Bifunctional Al-Doped Cobalt Ferrocyanide Nanocube Array for Energy-Saving Hydrogen Production via Urea Electrolysis

**DOI:** 10.3390/molecules28207147

**Published:** 2023-10-18

**Authors:** Xiafei Gao, Mengyue Gao, Xueping Yu, Xiaoyong Jin, Gang Ni, Juan Peng

**Affiliations:** State Key Laboratory of High-Efficiency Utilization of Coal and Green Chemical Engineering, College of Chemistry and Chemical Engineering, Ningxia University, Yinchuan 750021, Chinanigang@nxu.edu.cn (G.N.)

**Keywords:** hydrogen, urea oxidation, electronic structure, energy-saving, cobalt ferrocyanide

## Abstract

The very slow anodic oxygen evolution reaction (OER) greatly limits the development of large-scale hydrogen production via water electrolysis. By replacing OER with an easier urea oxidation reaction (UOR), developing an HER/UOR coupling electrolysis system for hydrogen production could save a significant amount of energy and money. An Al-doped cobalt ferrocyanide (Al-Co_2_Fe(CN)_6_) nanocube array was in situ grown on nickel foam (Al-Co_2_Fe(CN)_6_/NF). Due to the unique nanocube array structure and regulated electronic structure of Al-Co_2_Fe(CN)_6_, the as-prepared Al-Co_2_Fe(CN)_6_/NF electrode exhibited outstanding catalytic activities and long-term stability to both UOR and HER. The Al-Co_2_Fe(CN)_6_/NF electrode needed potentials of 0.169 V and 1.118 V (vs. a reversible hydrogen electrode) to drive 10 mA cm^−2^ for HER and UOR, respectively, in alkaline conditions. Applying the Al-Co_2_Fe(CN)_6_/NF to a whole-urea electrolysis system, 10 mA cm^−2^ was achieved at a cell voltage of 1.357 V, which saved 11.2% electricity energy compared to that of traditional water splitting. Density functional theory calculations demonstrated that the boosted UOR activity comes from Co sites with Al-doped electronic environments. This promoted and balanced the adsorption/desorption of the main intermediates in the UOR process. This work indicates that Co-based materials as efficient catalysts have great prospects for application in urea electrolysis systems and are expected to achieve low-cost and energy-saving H_2_ production.

## 1. Introduction

With increasing consumption of traditional fossil fuels and environmental pollution, it is important to find clean and sustainable energy sources. Hydrogen (H_2_) is an environmentally friendly alternative fossil fuel due to its high energy efficiency, sustainability, and zero carbon emissions [1]. Water electrolysis consists of an anodic oxygen evolution reaction (OER) and a cathodic hydrogen evolution reaction (HER), which is a promising and environmentally friendly method to produce hydrogen. However, the very slow and complex OER kinetics lead to greatly increased energy consumption and hydrogen prices, thus hindering its large-scale application. Urea wastewater is a rich and undeveloped energy source generated during industrial urea production and human metabolism. If urea is directly released into the environment, it decomposes into toxic substances, causing pollution and affecting water sources [2]. Urea electrolysis utilizes a urea oxidation reaction (UOR) to provide electrons as an anode. Pairing HER and UOR in urea electrolysis can be used for lower-energy H_2_ generation, which also reduces urea water pollution [3,4,5,6,7].

At present, platinum-based and ruthenium-based materials are the preferred catalysts for UOR in neutral and alkaline media [8,9,10]. However, they are expensive and cannot be applied in a large-scale process. Therefore, developing efficient and economical catalysts to achieve excellent UOR catalytic performance is of great significance. In recent years, transition metal-based nanomaterials have attracted great attention in electrocatalysis [11,12]. By studying the bifunctional catalytic ability of electrocatalysts for oxygen evolution reactions and urea oxidation reactions [13], it is found that transition metal-based nanomaterials are excellent UOR electrocatalysts due to their higher activity in an alkaline medium than precious metals [14,15,16]. Up to now, Ni hydroxides [17,18], oxides [19], sulfides [20,21,22], and phosphides [23,24] have been developed for UOR with commendable performance. For example, Tesfaye et al. [25] synthesized a carbon nanotube aerogel catalyst modified with Ni-Co bimetallic nanoparticles, which can significantly enhance the current density of UOR and lower the overpotential. Li et al. [26] successfully prepared Fe/N co-doped Ni_3_S_2_ and NiP_2_ heterostructures for efficient UOR. Due to the hierarchical structure of N-Fe-Ni_3_S_2_@NiP_2_/NF material, the large number of exposed active sites, and the doping effect of N and Fe, the material shows excellent electrocatalytic activity for UOR. Shen et al. [27] designed a spherical Co_3_S_4_ and Ni-Fe sulfide porous nanosheet array catalyst (NiFeCoSx@FeNi_3_) growing vertically on FeNi_3_ foam, which has excellent UOR and HER catalytic activity and stability in an alkaline solution. However, the UOR catalytic performance of these catalysts needs to be further improved. Hence, the development of non-noble metal-based catalysts for effective UOR and HER is a promising method to simplify the catalytic system and reduce manufacturing and energy costs.

It is well known that an electrocatalytic reaction is a multi-step reaction, including the adsorption/desorption process of reactants and products and the electron transfer that takes place on the surface of the catalyst. Therefore, catalysts largely depend on their surface electronic structure [28]. Heteroatom doping is a proof-of-concept way to regulate the electronic structure, thus boosting the catalytic activity to urea oxidation and hydrogen evolution activities of these nonprecious metal-based catalysts [29,30]. Heteroatom-doped catalysts can also be used as bifunctional catalysts to promote the catalytic performance of HER and UOR and realize energy-saving hydrogen production and degradation of urea-containing wastewater [14,31,32].

In this work, an Al-doped cobalt ferrocyanide (Al-Co_2_Fe(CN)_6_) nanocube array was prepared on nickel foam (Al-Co_2_Fe(CN)_6_/NF) by one-step in situ growth. The Al-Co_2_Fe(CN)_6_/NF exhibited outstanding bifunctional catalytic activities to both UOR and HER in alkaline media. Density functional theory (DFT) indicates that Co sites with Al doping to regulate electronic structure greatly contribute to the UOR activity of materials. The as-prepared urea electrolysis system combining UOR and HER only needed a cell voltage of 1.357 V to achieve 10 mA cm^−2^, which saved 11.2% electricity energy compared to that of traditional water splitting. This work not only provides a bifunctional electrocatalyst for cost-saving hydrogen production, but, more importantly, proposes a potential way of purifying urea-rich wastewater in the future.

## 2. Results and Discussion

### 2.1. Characterizations

Al-doped Co_2_Fe(CN)_6_ was prepared by in situ growth on nickel foam. The prepared catalyst was characterized by X-ray diffraction (XRD) patterns. As shown in Figure S1, three characteristic peaks appeared at 44.5°, 51.8° and 76.4°, corresponding to the (111), (200) and (220) crystal planes of Ni (PDF#04-0850), respectively. Three Bragg peaks emerge at 17.54°, 24.92°, and 35.59°, which are ascribed to the (200), (220), and (400) planes of Co_2_Fe(CN)_6_ (PDF #14-0291). After Al doping, the (200), (220), and (400) planes of Co_2_Fe(CN)_6_ were also observed in the patterns with a slight shift. The crystal structure of Al-Co_2_Fe (CN)_6_/NF is similar to that of Co_2_Fe(CN)_6_/NF, indicating that the doping of Al has a negligible effect on the crystal structure.

**Figure 1:**
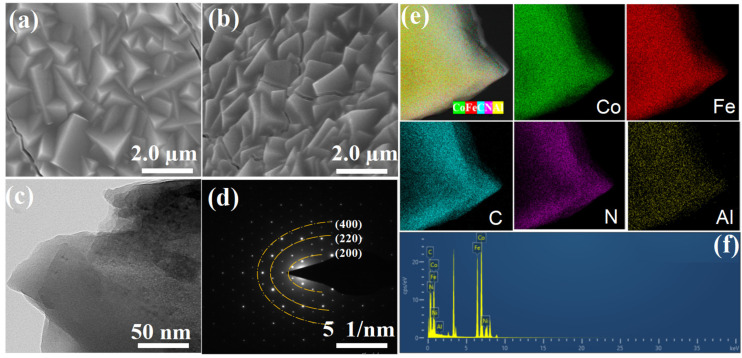
SEM images of (**a**) Co_2_Fe(CN)_6_/NF and (**b**) Al-Co_2_Fe(CN)_6_/NF; (**c**) TEM and (**d**) SAED images of Al-Co_2_Fe(CN)_6_/NF; (**e**) HAADF-HRTEM and corresponding elemental mapping images of Al-Co_2_Fe(CN)_6_/NF; (**f**) EDX spectrum of Al-Co_2_Fe(CN)_6_/NF.

The morphological features of Co_2_Fe(CN)_6_/NF and Al-Co_2_Fe(CN)_6_/NF were characterized by scanning electron microscopy (SEM) and transmission electron microscopy (TEM). As shown in Figure 1a,b and Figure S2, the morphology of Co_2_Fe(CN)_6_/NF and Al-Co_2_Fe(CN)_6_/NF are composed of many tightly bound nanocubes, indicating that the doping of aluminum elements almost does not change the morphology. The morphology of the composite is uniformly distributed. As shown in Figure 1c,d, a ring consisting of a large number of discrete spots can be observed in the map of constituency electron diffraction (SAED) of Al-Co_2_Fe(CN)_6_/NF, with three distinct rings corresponding to the (200), (220) and (400) planes of Co_2_Fe(CN)_6_/NF, which is consistent with the XRD, indicating that the composite has a polycrystalline shape. As shown in Figure 1e, for the TEM element map of Al-Co_2_Fe(CN)_6_/NF, all Co, Fe, C, N, and Al elements are evenly distributed in the sample, and from the EDX spectrum of Al-Co_2_Fe(CN)_6_/NF in Figure 1f, doped Al atoms constituted only 2% of the atoms in the sample.

**Figure 2:**
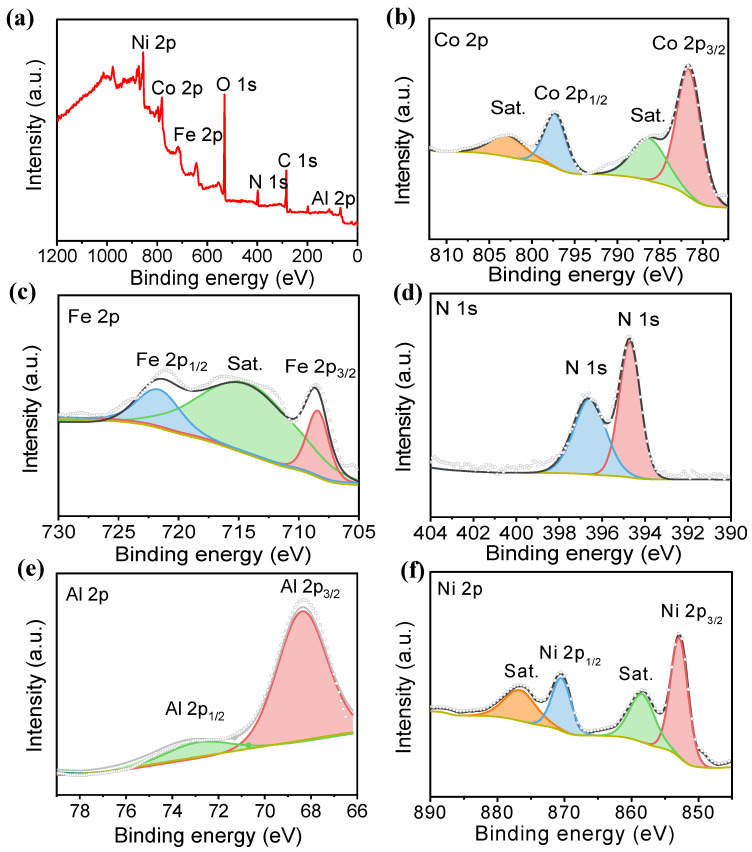
(**a**) XPS survey spectra of Al-Co_2_Fe(CN)_6_/NF; (**b**) Co 2p spectrum; (**c**) Fe 2p spectrum; (**d**) N 1s spectrum; (**e**) Al 2p spectrum; (**f**) Ni 2p spectrum.

The surface chemical composition and valence state of the Al-Co_2_Fe(CN)_6_/NF were monitored by X-ray photoelectron spectroscopy (XPS). As shown in Figure 2a, the XPS survey spectra showed that Co, Fe, N, Al, and Ni elements exist on the catalyst surface. In the Co 2p spectrum (Figure 2b), two main peaks were found at 781.1 eV and 797.1 eV, corresponding to Co 2p_3/2_ and Co 2p_1/2_ of cobalt hydroxide [33]. As shown in Figure 2c, there were two main peaks at 708.5 eV and 721.1 eV in the Fe 2p spectrum, corresponding to characteristic bonds of Fe 2p_3/2_ and Fe 2p_1/2_ of iron hydroxide. Figure 2d showed that the N 1s XPS spectrum was concentrated at 398.5 eV and 400.0 eV, indicating the presence of pyridine-N and pyrrole-N in the Al-Co_2_Fe(CN)_6_. In Figure 2e, binding energies at 73.54 eV and 67.48 eV in Al 2p spectrum accompanied by satellite peaks were observed, which belonged to the Al 2p_1/2_ and Al 2p_3/2_ orbits, respectively. The high-resolution Ni 2p spectrum in Figure 2f has two peaks, at 871.97 eV and 854 eV, corresponding to Ni 2p_3/2_ and Ni 2p_1/2_, respectively, indicating the presence of nickel hydroxide [34].

### 2.2. Electrochemical Catalytic Performances

**Figure 3:**
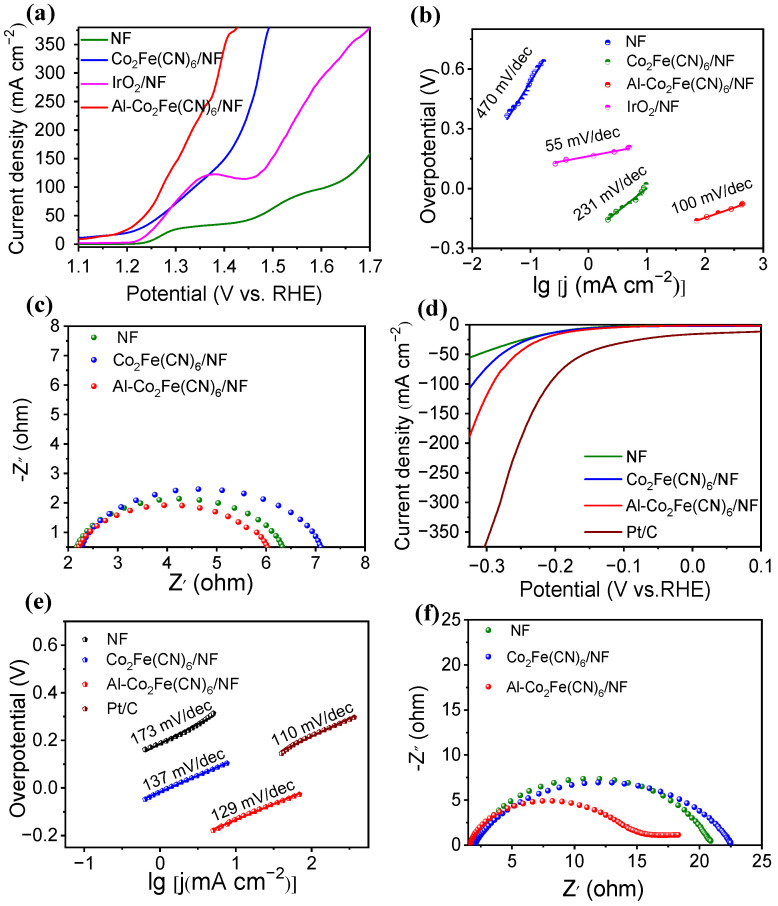
(**a**) The polarization curves of catalysts in Ar-saturated 1.0 M KOH with 0.5 M urea; (**b**) Tafel slope diagram for UOR; (**c**) EIS; (**d**) the polarization curves of catalysts in Ar-saturated 1.0 M KOH; (**e**) corresponding Tafel slope diagram for HER; (**f**) EIS diagram.

The electrocatalytic activity to UOR was performed in 1.0 M potassium hydroxide aqueous solution containing 0.5 M urea. The synthetic parameters for the Al-Co_2_Fe(CN)_6_/NF were optimized according to their catalytic performance by controlling the reaction times, Co^2+^ and Al^3+^ concentration, and the type of doped elements. Figures S3 and S4 show that the Al-Co_2_Fe(CN)_6_/NF exhibited the best catalytic property to UOR under optimal synthetic conditions at 24 h of reaction time; concentration of Co^2+^ and Al^3+^ at 0.75 M and 1 mM, respectively; and aluminum doping. As shown in Figure S5, the Al-Co_2_Fe(CN)_6_/NF composite exhibited the highest current response in the 1.0 M KOH electrolyte solution consisting of 0.5 M urea. Therefore, the catalytic reaction was performed in a 1.0 M KOH electrolyte solution consisting of 0.5 M urea. Linear scanning voltammetry (LSV) curves of the Al-Co_2_Fe(CN)_6_/NF, Co_2_Fe(CN)_6_/NF, and NF are shown in Figure 3. The Al-Co_2_Fe(CN)_6_/NF shows high activity to UOR, which only needs a potential of 1.272 V (vs. RHE) at a current density of 100 mA, much smaller than that of NF (1.61 V vs. RHE) and Co_2_Fe(CN)_6_/NF (1.339 V vs. RHE). Moreover, the potential of UOR for Al-Co_2_Fe(CN)_6_/NF is obviously lower than that to OER (1.440 V vs. RHE), resulting in energy-savings for hydrogen production. The UOR activity for Al-Co_2_Fe(CN)_6_/NF was comparable to other similar electrocatalysts in Table S1 (Supplementary Information). The results demonstrated that Al-Co_2_Fe(CN)_6_/NF possesses a satisfactory catalytic performance to UOR.

The Tafel slope values were obtained to study the reaction kinetics according to the LSV curves. As shown in Figure 3b, the Tafel slopes of Al-Co_2_Fe(CN)_6_/NF, Co_2_Fe(CN)_6_/NF, NF, and IrO_2_ to UOR are 100 mV/dec, 231 mV/dec, 470 mV/dec and 55 mV/dec, respectively. Among these, Al-Co_2_Fe(CN)_6_/NF has a smallest Tafel slope value, suggesting a fast electron transport in the electrochemical urea oxidation process. The excellent kinetic property of the Al-Co_2_Fe (CN)_6_/N during the UOR process was further reflected by the electrochemical impedance (EIS) test in 1.0 M KOH with 0.5 M urea. As shown in Figure 3c, the charge transfer resistance of Al-Co_2_Fe(CN)_6_/NF is 6.03 Ω, which is obviously lower than those of Co_2_Fe(CN)_6_/NF (6.39 Ω) and NF (7.18 Ω). This result proved that Al-Co_2_Fe(CN)_6_/NF had higher conductivity and faster electron transfer characteristics, which can greatly promote UOR performance.

In order to prove the superiority of the material’s catalytic activity, the HER catalytic activities of Al-Co_2_Fe(CN)_6_/NF, Co_2_Fe(CN)_6_/NF, NF, and the commercial Pt/C were also explored in the Ar-saturated 1.0 m KOH electrolyte. As shown in Figure 3d, the Al-Co_2_Fe(CN)_6_/NF demonstrated an overpotential of 169 mV (vs. RHE) at a current density of 10 mA/cm^2^, which is clearly lower than those of NF (188 mV vs. RHE) and Co_2_Fe(CN)_6_/NF (184 mV vs. RHE), confirming the enhanced activity of Al-Co_2_Fe (CN)_6_/NF. However, the HER activity of Al-Co_2_Fe(CN)_6_/NF is still poorer than that of commercial Pt/C. The polarization curves of the Al-Co_2_Fe(CN)_6_/NF to HER in different electrolyte solutions of 1.0 M KOH, 1.0 M KOH with 0.33 M urea, and 1.0 M KOH with 0.5 M urea are present in Figure S5b. The HER activity of Al-Co_2_Fe(CN)_6_/NF is essentially identical in 1.0 M KOH and 1.0 M KOH with 0.5 M urea. This result confirmed the urea has negligible electrolyte effects on the HER activity of Al-Co_2_Fe(CN)_6_/NF.

To further analyze the kinetics of the catalysts to HER, the Tafel slopes were demonstrated in Figure 3e. The Tafel slopes of Al-Co_2_Fe(CN)_6_/NF, Co_2_Fe(CN)_6_/NF, NF, and Pt/C for HER are 129 mV/dec, 137 mV/dec, 137 mV/dec, 173 mV/dec and 110 mV/dec, respectively. In contrast to Co_2_Fe(CN)_6_/NF and NF, Al-Co_2_Fe (CN)_6_/NF has the smallest Tafel slope value. This verified the facilitated HER kinetics of Al-Co_2_Fe (CN)_6_/NF and was consistent with the Volmer–Heyrovsky mechanism in the alkaline media. The electron transfer kinetics during the HER process can be reflected by the EIS of the Al-Co_2_Fe(CN)_6_/NF in 1.0 M KOH with 0.5 M urea. According to Figure 3f, the charge transfer resistance of Al-Co_2_Fe(CN)_6_/NF (15.01 Ω) is smaller than those of Co_2_Fe(CN)_6_/NF (21.05 Ω) and NF (22.60 Ω). This result indicates that Al Co_2_Fe (CN) _6_/NF has a fast electron transfer rate and high conductivity, which contributes greatly to the HER activity. The electric double-layer capacitance (Cdl) reflects the value of the electrochemical active surface area (ECSA). The CVs of Al-Co_2_Fe(CN)_6_/NF, Co_2_Fe(CN)_6_/NF, and NF in 1.0 M KOH with 0.5 M urea at 10–110 mV/s sweep are shown in Figure S6a–c, respectively. Linear fitting diagrams of the current density difference (Δj) and the sweep speed were obtained for Al-Co_2_Fe(CN)_6_/NF, Co_2_Fe(CN)_6_/NF, and NF, as shown in Figure S6d. The capacitance value (Cdl) of Al-Co_2_Fe(CN)_6_/NF (6.255 mF/cm^2^) is larger than that of the Co_2_Fe(CN)_6_/NF (2.745 mF/cm^2^) and NF electrodes (0.695 mF/cm^2^). This result indicated that the Al-Co_2_Fe(CN)_6_/NF had a high ECSA, which may offer more active sites for HER.

### 2.3. Whole-Urea Electrolysis

**Figure 4:**
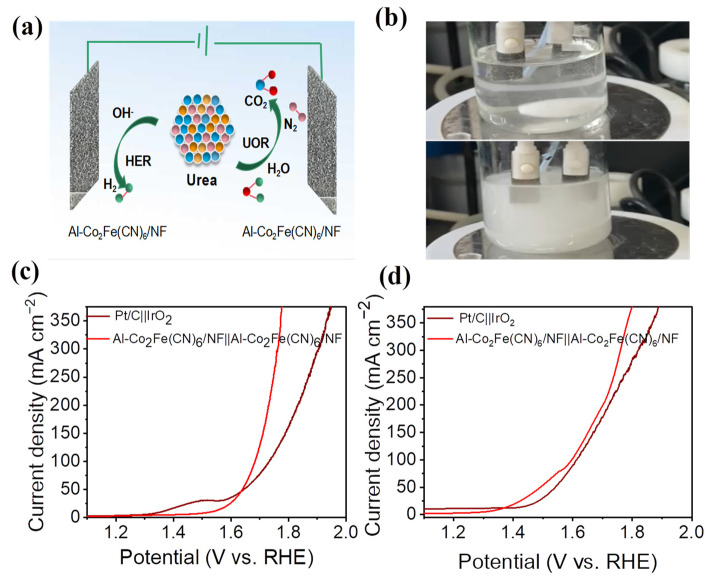
(**a**) Simulation diagram for HER||UOR system using Al-Co_2_Fe(CN)_6_/NF as both cathode and anode; (**b**) photograph of HER||OER system, and the electrolyte is 1 M KOH (top); HER||UOR system (down), the electrolyte is 1.0 M KOH with 0.5 M urea; (**c**) the polarization curves of Pt/C||IrO2 and Al-Co_2_Fe(CN)_6_/NF||Al-Co_2_Fe(CN)_6_/NF for overall water electrolysis in 1.0 M KOH; (**d**) the polarization curves of Pt/C||IrO_2_ and Al-Co_2_Fe(CN)_6_/NF||Al-Co_2_Fe(CN)_6_/NF for whole-urea electrolysis in 1.0 M KOH with 0.5 M urea.

Considering the good electrocatalytic performance of the Al-Co_2_Fe(CN)_6_/NF electrodes to both HER and UOR, a whole-urea electrolysis system was developed using Al-Co_2_Fe(CN)_6_/NF as an anode and a cathode, respectively. Figure 4a illustrates the coupling HER||UOR system. Figure 4b is the optical image of the HER||OER system and the HER||UOR system for hydrogen generation. The generated bubbles are more obvious in the HER||UOR system than in the HER||OER system, indicating that the catalyst has a better catalytic performance for the UOR. From the polarization curve in Figure 4c, the current density of the HER||OER system with Al-Co_2_Fe(CN)_6_/NF as the electrode reached 10 mA cm^–2^ at a cell voltage of 1.52 V. However, the HER||UOR system only needed a cell voltage of 1.36 V to achieve a current density of 10 mA cm^–2^, as shown in Figure 4d, which is much smaller than the traditional water electrolysis system. At the same time, under the cell voltage of 1.49 V (corresponding to the current density of 50 mA cm^–2^), compared with the water electrolysis system, the current density of the water–urea system is increased by about 6.35 times. This proves that replacing OER with UOR is an energy-saving strategy for hydrogen production. In addition, in the same HER||UOR coupling system, the overall electrocatalytic performance of Al-Co_2_Fe(CN)_6_/NF is even better than that of a noble metal-based catalyst, that is, at a current density of 50 mA cm^–2^, the battery voltage is 1.492 V, while the Pt/C‖RuO_2_ is 1.542 V.

### 2.4. Stability of the Catalyst

**Figure 5:**
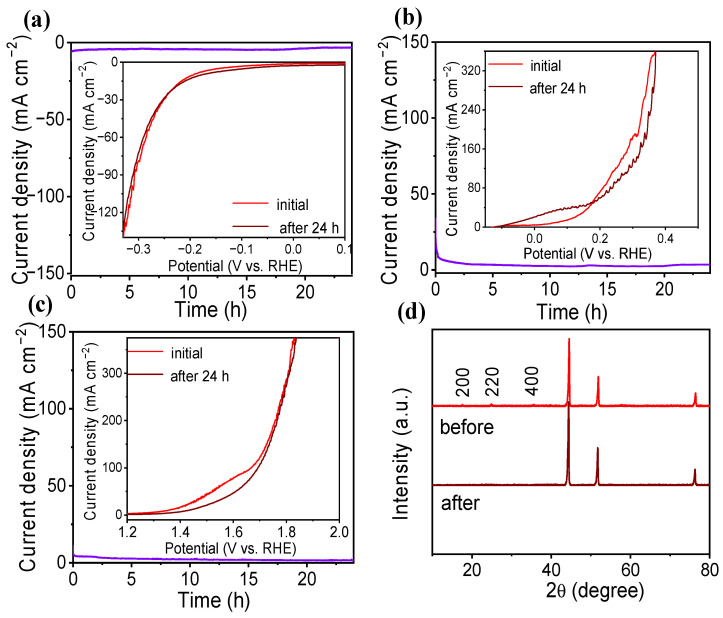
(**a**) Durability test of Al-Co_2_Fe(CN)_6_/NF to HER (Purple lines shows the stability test curve). The inset shows the polarization curves of Al-Co_2_Fe(CN)_6_/NF before and after 24 h electrolysis; (**b**) UOR durability test of Al-Co_2_Fe(CN)_6_/NF. The inset shows the polarization curves of Al-Co_2_Fe(CN)_6_/NF before and after the 24 h electrolysis; (**c**) durability test for whole-urea electrolysis using Al-Co_2_Fe(CN)_6_/NF || Al-Co_2_Fe(CN)_6_/NF. (**d**) XRD patterns of Al-Co_2_Fe(CN)_6_/NF before and after continuous electrocatalysis for 24 h.

Under the current density of 10 mA cm^−2^, the stability of Al-Co_2_Fe(CN)_6_/NF to HER, UOR, and whole-urea electrolysis was tested, as shown in Figure 5. From Figure 5a, the current density was maintained for continuous 24 h electrolysis. The polarization curves also did not changed before and after 24 h electrolysis, suggesting the high HER stability of Al-Co_2_Fe(CN)_6_/NF. It can be seen in Figure 5b that Al-Co_2_Fe(CN)_6_/NF also has good stability for UOR. As shown in Figure 5c, the current density decreased slightly but tended to be stable. The LSV was basically the same before and after the reaction, which proved that the Al-Co_2_Fe(CN)_6_/NF assembled into an HER||UOR cell still had good stability. According to the XRD pattern in Figure 5d, there is no obvious change for Al-Co_2_Fe(CN)_6_/NF before and after urea electrolysis, indicating that the Al-Co_2_Fe(CN)_6_/NF possesses a stable chemical structure.

**Figure 6:**
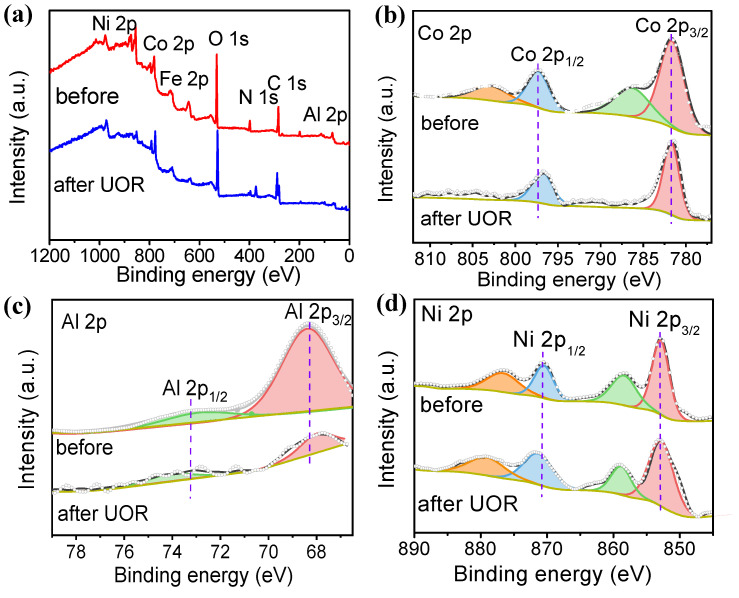
(**a**) XPS survey spectra of Al-Co_2_Fe(CN)_6_/NF; (**b**) Co 2p spectrum; (**c**) Al 2p spectrum; (**d**) Ni 2p spectrum before and after 24 h electrolysis.

Figure 6a is an XPS spectrum of Al-Co_2_Fe(CN)_6_/NF before and after 24 h electrolysis. As shown in the figure, by observing the full spectrum of XPS, Al-Co_2_Fe(CN)_6_/NF is mainly composed of Co, Fe, C, N, and Al, which is consistent with the result of element mapping, and the peak of Ni is generated by the NF substrate. As shown in Figure 6b–d, the peaks shifted slightly before and after the reaction, while XPS moved to the right, indicating high prices and higher ratios of Ni^3+^/Ni^2+^, Co^3+^/Co^2+^, and Fe^3+^/ Fe^2+^. No CoOOH, FeOOH, and NiOOH peaks were observed during 24 h electrolysis, suggesting that Co^2+^, Fe^2+^, and Ni^2+^ were not fully oxidized during catalysis, which is different from the mechanism reported for other nickel-based catalysts [35,36].

### 2.5. Catalytic Mechanisms

**Figure 7:**
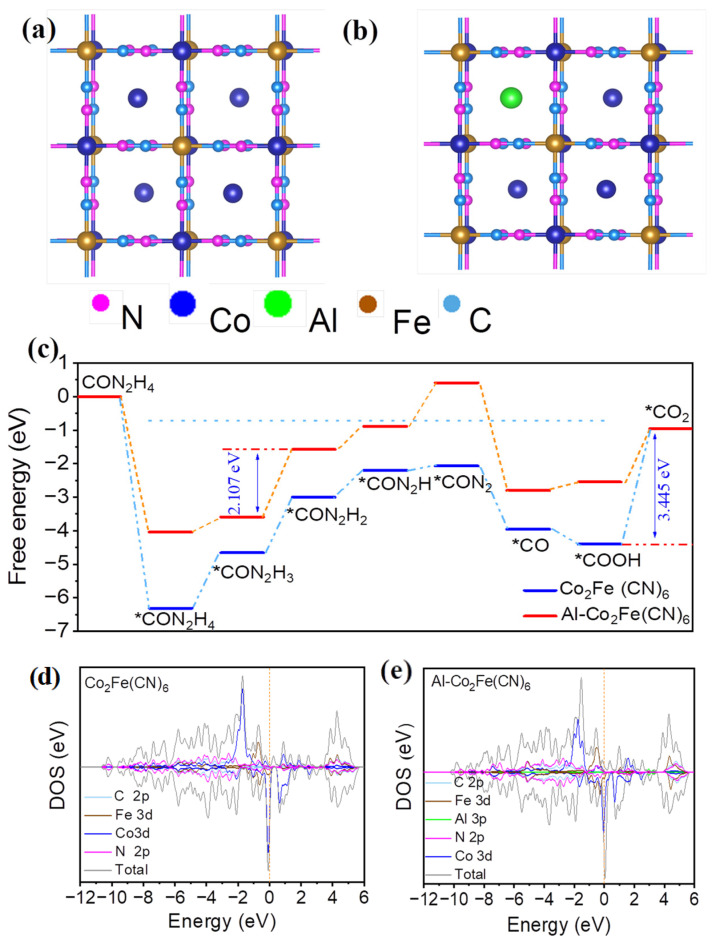
(**a**) Schematic diagram of the structure of Co_2_Fe(CN)_6_; (**b**) schematic diagram of the structure of Al-Co_2_Fe(CN)_6_; (**c**) the Gibbs free energy changes in the UOR process; (**d**) DOS of the Co_2_Fe(CN)_6_; (**e**) DOS of the Al-Co_2_Fe(CN)_6_.

The UOR process has a 6e transfer reaction, which establishes a mature reaction mechanism and mature proportional relationship for each reaction intermediate. It has been reported that there are many substitution mechanisms on the surface of NiOOH [37]. This has been extensively validated in other multi-electron transfer reactions. The schematic diagram of Co_2_Fe(CN)_6_ is shown in Figure 7a; the whole structure is a square structure composed of four small units, which are regularly distributed. The doping of Al may replace the position of partial Co^2+^, so it is speculated that the structural diagram of Al-Co_2_Fe(CN)_6_ should be shown in Figure 7b. In this structure, the Fe atom is at the various vertices of the quartet, the Co atom is at the center of the scaffold, and this stable structure gives it better UOR performance.

In order to further explore the reason why catalysts improve the activity of UOR, a density functional theory (DFT) calculation was carried out to reveal the potential catalytic mechanism. The typical UOR reaction path is as follows:

$${*}_{CO({NH}_{2}{)}_{2}\to }{*}_{CO(NH\cdot {NH}_{2})\to }{*}_{CO(NH\cdot NH)\to }{*}_{CO(NH\cdot N)\to }{*}_{CO(N_{2})\to }{*}_{CO(OH)\to }{*}_{CO(OH\cdot OH)\to }{*}_{COO}$$

where attachment of the *COO intermediate is the rate determination step [38,39]. The rate determination step (RDS) is to desorb the last *COOH intermediate from the active site to form *COO. However, due to the strong combination of active sites and *COOH intermediates, high energy is required for the reaction (ΔG = 3.445 eV), resulting in low catalyst activity. However, after the introduction of Al, the decisive step (RDS) of the whole reaction is that the intermediate *CON_2_H_3_ desorbs from the active center to form *CON_2_H_2_, the required ΔG for the reaction is reduced from 3.445 eV to 2.107 eV, and the required energy is significantly reduced, as shown in Figure 7c. Therefore, by calculating the Gibbs free energy of each intermediate, it is found that with the introduction of Al, the activation energy of the intermediate decreases, making it easier for the reaction to occur. The experimental and theoretical results show that aluminum doping is indeed beneficial to the catalytic reaction of UOR.

By calculating the DOS (density of states) of the catalyst, we further explain the potential reasons for the catalyst’s improved performance. As shown in Figure 7d,e, because the electronic states near the Fermi level are mainly provided by the d orbitals of Co and Fe atoms, and Co and Fe are active species, the d-band centers of Co and Fe in the Co_2_Fe(CN)_6_ catalyst are −1.416 eV and −0.599 eV, and those in the Al-Co_2_Fe(CN)_6_ catalyst are −1.406 eV and −0.369 eV. The introduction of Al changes the electrons around Co and Fe, adjusts the electronic structure, and makes the electronic state more active, which is beneficial to the adsorption of catalysts and intermediates and promotes the reaction.

## 3. Experiments

### 3.1. Materials and Chemicals

Ni foam was purchased from Tianjin Aiweixin Chemical Technology Co., Ltd. (Tianjin, China); CoCl_2_·6H_2_O (analytical reagent, AR) and Al(NO_3_)_3_ (AR) were purchased from Sinopharm Chemical Reagent. CH_3_CH_2_OH, KOH(AR), urea (AR), C_6_H_5_Na_3_O_7_·2H_2_O (AR), and K_3_[Fe(CN)_6_] (AR) were purchased from Tianjin Damao Chemical Reagent Factory. Polyvinylpyrrolidone (PVP) was purchased from Macklin. Nafion (5wt%) was purchased from The United States DuPont; Pt/C and IrO_2_ were from Aladdin.

### 3.2. Preparation of the Al-Co_2_Fe(CN)_6_/NF Electrode

The nickel foam was first cut into 4 cm × 4 cm pieces; washed with acetone, ethanol, and deionized water for 30 min and then dried for future use. In total, 0.5000 g of PVP, 0.1784 g of CoCl_2_·6H_2_O, 0.3088 g of C_6_H_5_Na_3_O_7_·2H_2_O, and 0.0375 g of Al(NO_3_)_3_ were added in 150 mL deionized water, stirring at room temperature to form aqueous solution A. Then, nickel foam was added to solution A. Additionally, 0.0823 g of K_3_[Fe(CN)_6_] was dissolved in 100 mL of deionized water to form aqueous solution B. Subsequently, solutions A and B were mixed, sealed, and stirred for 24 h. Finally, the Al-Co_2_Fe(CN)_6_/NF was washed thoroughly with deionized water and dried overnight. The Co_2_Fe(CN)_6_/NF was obtained using the procedure outlined above but without adding Al(NO_3_)_3_.

### 3.3. Electrochemical Measurements

The electrochemical performance of the UOR and HER was tested in a three-electrode system on an electrochemical workstation (CHI 920D). As-prepared Al-Co_2_Fe(CN)_6_/NF was used as a working electrode, a saturated calomel electrode (SCE) was used as the reference electrode, and a graphite rod was used as the counter-electrode. All potentials reported in this work reference the reversible hydrogen electrode (RHE) according to E (vs. RHE) = E(vs. SCE) + 0.0591 × pH + 0.242. Hydrogen overpotential and oxygen overpotential are calculated using the formulae $\eta =0-E\left(vs.RHE\right)$ and $\eta =E\left(vs.RHE\right)-1.23V$, respectively.

The cyclic voltammogram (CV) was recorded at scan rates of 10−110 mV s^−1^. The LSV curve was recorded at a scan rate of 5 mV s^−1^. The iR compensation level is 100%. Electrochemical impedance spectroscopy (EIS) tests were measured over a frequency range from 10^5^ to 10^−2^ Hz with an amplitude of 5 mV. The electrochemical double capacitance (Cdl) was calculated from the CV curve at different sweep speeds (10**–**110 mV s^−1^). The i-t curves were obtained to test stability.

### 3.4. DFT

The VASP mode was employed to obtain the DFT calculations [40]. Exchange and correlation potentials were modeled by selecting the GGA-PBE function [41]. The DFT-D3 function also considers weak van der Waals interactions [42]. The cutoff energy value of plane waves was 400 eV. We selected gamma points in the Brillouin zone for integration. In the iterative solution of the Kohn Sham equation, the total energy of the system converged to 10^−5^ eV. After geometric optimization, the force on each atom was reduced to 0.05 eV/Å. The Gibbs free energy is defined as G = E_tot_ + E_ZPE_ − TS, where E_tot_, E_ZPE_, and TS are total energy, zero-point energy, and entropy of the system.

## 4. Conclusions

In conclusion, an Al-Co_2_Fe(CN)_6_ nanocube array is grown in situ on nickel foam (Al-Co_2_Fe(CN)_6_/NF) by one-step in situ growth. Due to the unique nanocube array structure and regular electronic structure of Al-Co_2_Fe(CN)_6_, the prepared Al-Co_2_Fe(CN)_6_/NF catalyst showed excellent catalytic activity and long-term stability for HER and UOR. During the electrolysis process, the chemical structure and valence state of Co in Al-Co_2_Fe(CN)_6_ catalysis is not turned into Co hydroxide derivatives. Using a combination of experiments and DFT calculation, a more favorable UOR pathway at Al-Co_2_Fe(CN)_6_/NF is proposed. The DFT results show that the doping of Al can optimize the electronic structure, thus improving the adsorption and significantly enhancing the catalytic activity to UOR. Al-Co_2_Fe(CN)_6_/NF was used as the cathode and anode, and an energy-saving two-electrode system for hydrogen generation was constructed. The Al-Co_2_Fe(CN)_6_/NF requires less electric power and reduces the urea content of wastewater. Consequently, this work will open a way for the development of sustainable energy conversion by combining hydrogen production with urea wastewater treatment.

## Data Availability

All data in this study can be found in public databases and Supplementary Information, as described in the Experiments section (Section 3).

## Supplementary Materials

The following supporting information can be downloaded at https://www.mdpi.com/article/10.3390/molecules28207147/s1. References [43,44,45,46,47,48,49,50,51] are cited in the supplementary materials.
