# Supplementary material for: Bifunctional Al-Doped Cobalt Ferrocyanide Nanocube Array for Energy-Saving Hydrogen Production via Urea Electrolysis
Source: Molecules. 2023 Oct 18;28(20):7147. doi: 10.3390/molecules28207147 (PMC10608971; doi:10.3390/molecules28207147)
Supplement: Supplementary file 1 [file molecules-28-07147-s001.zip › molecules-2624160-supplementary.pdf]

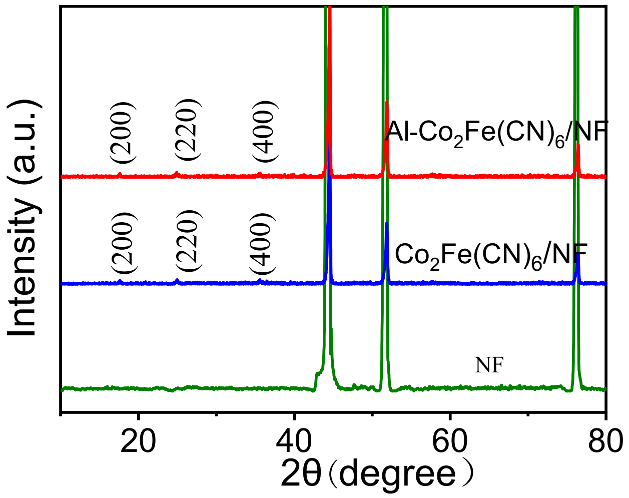

Figure S1. XRD patterns of NF, $Co_2Fe(CN)_6$/NF and Al-$Co_2Fe(CN)_6$/NF.

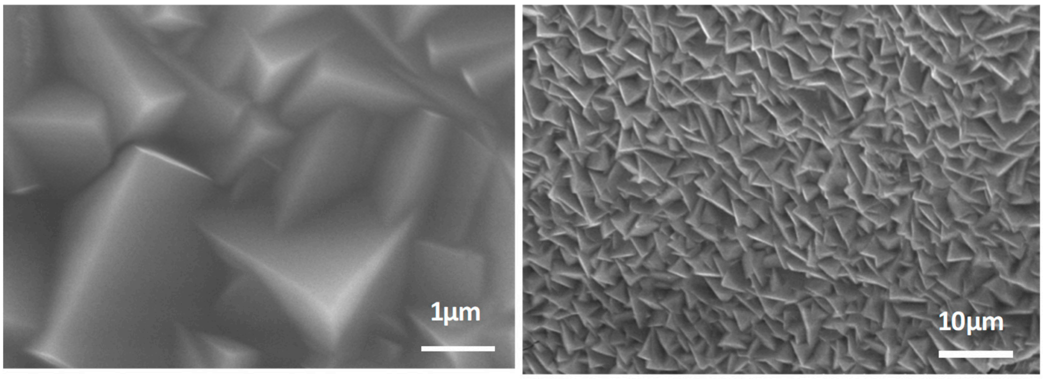

Figure S2. SEM images of Al-$Co_2Fe(CN)_6$/NF.

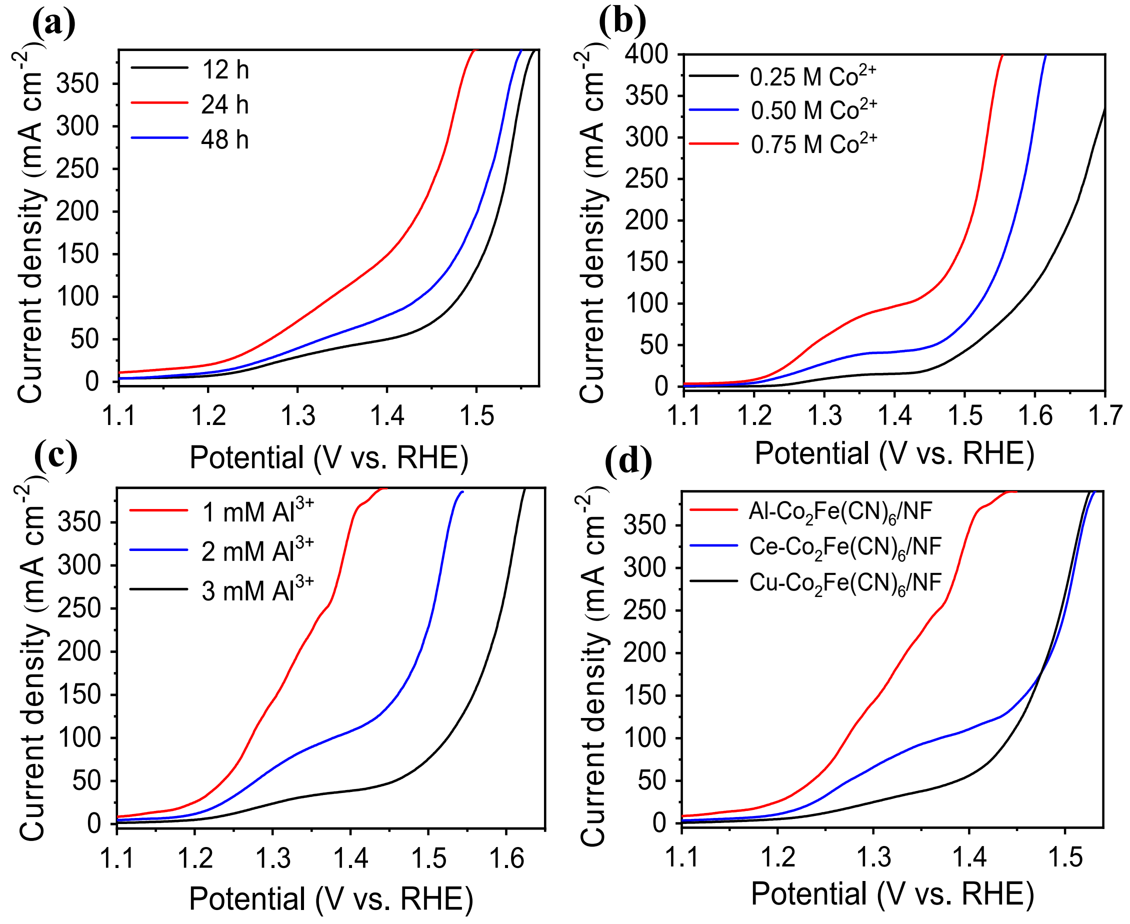

Figure S3. (a) The polarization curves of UOR of Al-$Co_2Fe(CN)_6$/NF at different stirring times.(b) The polarization curves of Al-$Co_2Fe(CN)_6$/NF with different Co^{2+} concentration. (c) The polarization curves of UOR of Al-$Co_2Fe(CN)_6$/NF with different Al^{3+} concentrations. (d) The polarization curves of UOR of $Co_2Fe(CN)_6$/NF doped with different metals.

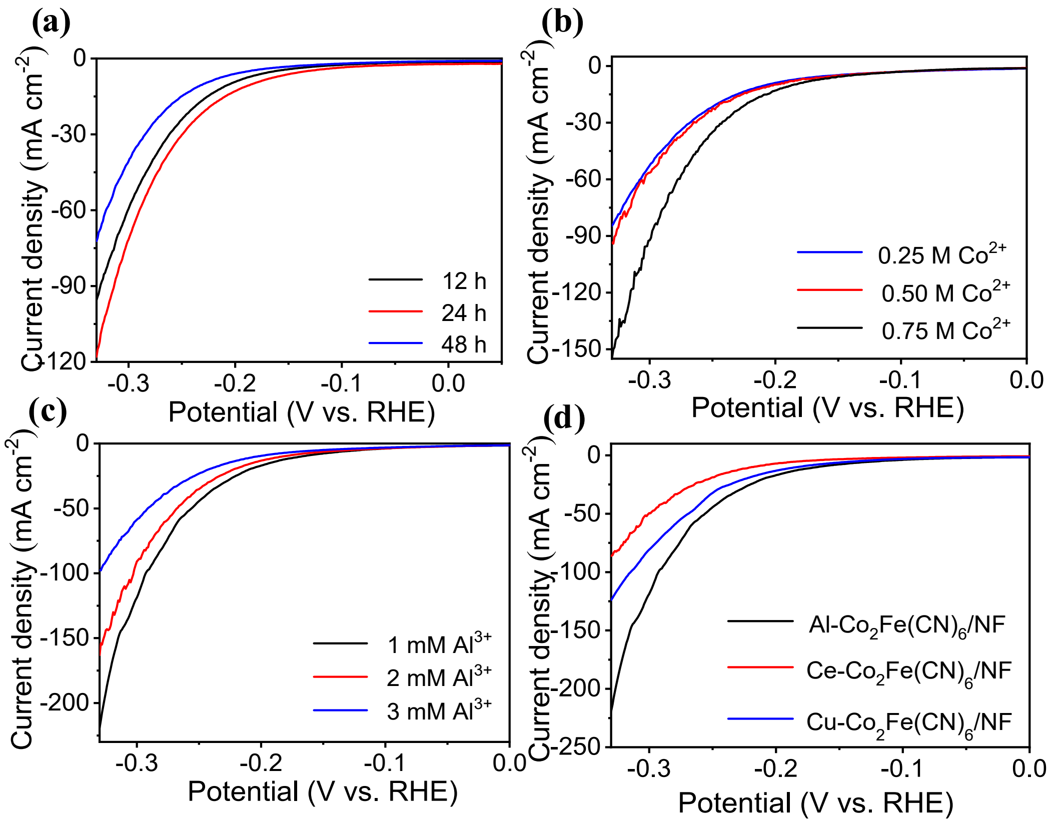

Figure S4. (a) The polarization curves of HER of Al-$Co_2Fe(CN)_6$/NF at different stirring times. (b) The polarization curves of HER of Al-$Co_2Fe(CN)_6$/NF with different Co^{2+} concentration. (c) The polarization curves of HER of Al-$Co_2Fe(CN)_6$/NF with different Al^{3+} concentrations. (d) The polarization curves of HER of $Co_2Fe(CN)_6$/NF doped with different metals.

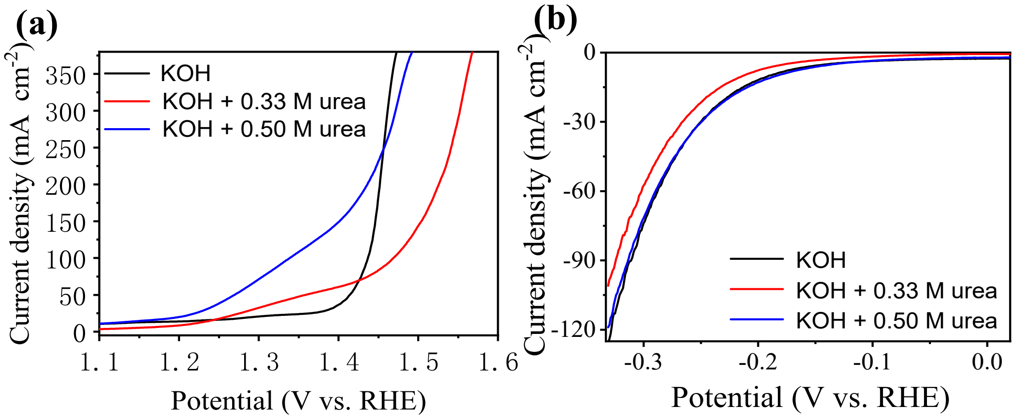

Figure S5. (a) Polarization curves of UOR of Al-$Co_2Fe(CN)_6$/NF in electrolyte solutions under different conditions. (b) The polarization curves of HER of Al-$Co_2Fe(CN)_6$/NF in electrolyte solutions under different conditions.

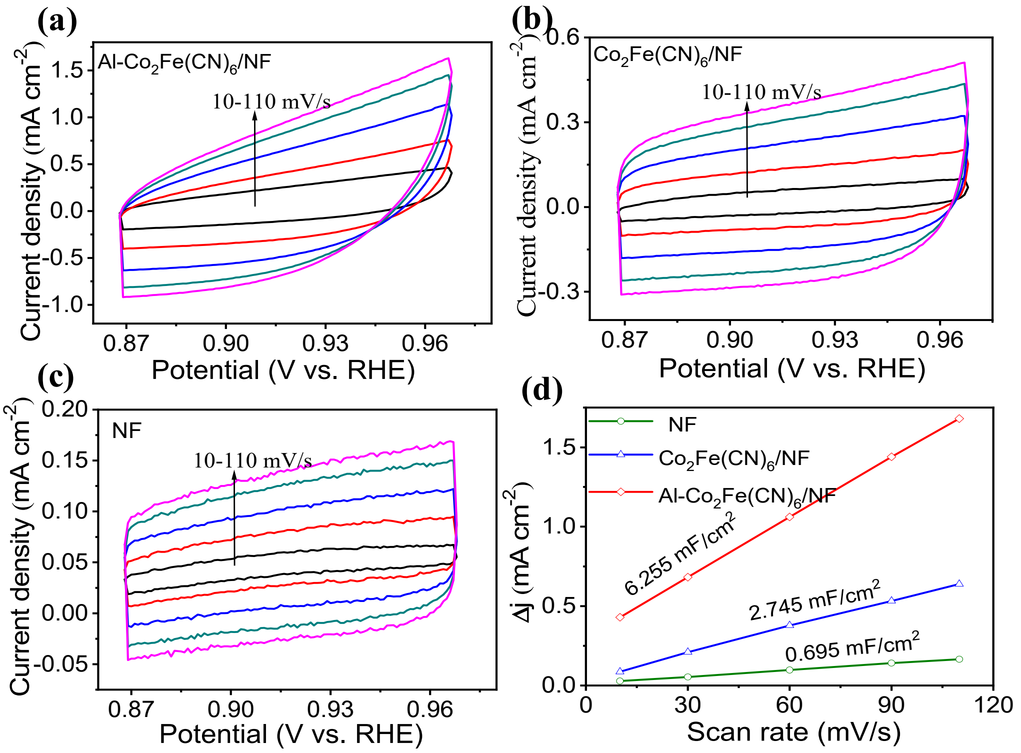

Figure S6. Cyclic voltammetry curves obtained at a sweep rate of 10-110 mV/s. (a) Al-$Co_2Fe(CN)_6$/NF. (b) $Co_2Fe(CN)_6$/NF. (c) NF. (d) Linear fitting of the capacitive current densities Δj against the CV scan rates.

Table S1. Comparison of the activities of different catalysts for UOR

Catalyst	Electrolyte	Potential (V vs. RHE) for 100 mA cm-2	Current density (mA cm-2) @ 1.4V	Catalyst support	Reference
Al-$Co_2Fe(CN)_6$	1M KOH+ 0.5M Urea	1.27	~370	Nickel foam	**This work**
$Ni_2Fe(CN)_6$	1M KOH+ 0.33 M Urea	1.35	255	Nickel foam	[43]
CeO2@CoFe2O4	1M KOH+ 0.5M Urea	1.41	96	Nickel foam	[44]
CoFe LDH/MOF	1M KOH+ 0.33 M Urea	1.57	5	CC	[45]
CoFe	1M KOH+ 0.33 M Urea	1.37	135	Nickel foam	[46]
O-NiMoP	1M KOH+ 0.5M Urea	1.41	~95	Nickel foam	[24]
Carbon/Ni-Fe	1M KOH+ 0.33M Urea	1.39	80	Nickel foam	[47]
MnO2/$MnCo_2O_4$	1M KOH+ 0.5M Urea	1.33	270	Nickel foam	[48]
Ni(OH)2	1M KOH+ 0.3 M Urea	1.44	~85	Nickel foam	[49]

Fe-O-P	1M KOH+ 0.5M Urea	1.39	~140	Nickel foam	[50]
MiMoO-Ar	1M KOH+ 0.5M Urea	1.42	~65	Nickel foam	[51]
